# The threshold of an intra oral scanner to measure lesion depth on natural unpolished teeth

**DOI:** 10.1016/j.dental.2022.06.022

**Published:** 2022-08

**Authors:** Polyvios Charalambous, Saoirse O’Toole, Rupert Austin, David Bartlett

**Affiliations:** a King’s College London, Faculty of Dentistry, Oral and Craniofacial Sciences, Guy’s Hospital, Tower wing, London SE1 9RT, UK; b Centre for Oral, Clinical & Translational Sciences, King’s College London, Faculty of Dentistry, Oral and Craniofacial Sciences, Guy’s Hospital, Tower wing, London SE1 9RT, UK

**Keywords:** Tooth wear, Dental enamel, Digital dentistry, Surface registration, Diagnostic imaging, Surface metrology

## Abstract

**Objectives:**

To investigate the threshold and accuracy of intraoral scanning in measuring freeform human enamel surfaces.

**Methods:**

Software softgauges, ranging between 20 and 160 µm depth, were used to compare four workflow analysis techniques to measure step height on a freeform surface; with or without reference areas and in combination with surface-subtraction to establish which combination produced the most accurate outcome. Having established the optimum combination, 1.5 mm diameter, individual depths ranging from 11 to 81 µm were created separately on 14 unpolished human enamel samples and then scanned with gold standard laboratory optical profilometry (NCLP, TaiCaan Technologies™, XYRIS2000CL, UK) and a clinical intraoral scanner (TrueDefinition™, Midmark Corp., USA). The sequence of surface registration and subtraction determined from the softgauges was used to measure step height on natural human enamel surfaces. Step heights (μm) were compared using two-way ANOVA with post-hoc Bonferroni (p < 0.05) and Bland-Altman analyses.

**Results:**

Software differences were significantly reduced from − 29.7 to − 32.5% without, to − 2.4 to − 3.6% with reference areas (p < 0.0001) and the addition of surface-subtraction after registration reduced this further to 0.0 to − 0.3% (p < 0.0001). The intraoral scanner had a depth discrimination threshold of 73 µm on unpolished natural enamel and significant differences (p < 0.05) were observed compared to NCLP below this level.

**Significance:**

The workflow of combining surface-registration and subtraction of surface profiles taken from intraoral scans of freeform unpolished enamel enabled confident measurement of step height above 73 µm. The limits of the scanner is related to data capture and these results provide opportunities for clinical measurement.

## Highlights

• Surface-registration and subtraction of profiles from free form surfaces improved accuracy in measuring surface change.
• The threshold for the intraoral scanner was ≥ 73 µm step height natural enamel lesions.
• The intra-oral scanner demonstrated inferior resolution than profilometry.

## 1 Introduction

Change on non-flat, freeform surfaces presents challenges for measurement of Z height using profilometry [1]. Freeform surface metrology refers to quantifying specific features on a point cloud mesh produced from optical scans of complex geometrical surfaces, such as seen on teeth. Over the last few years, there have been significant advances in software and hardware of surface metrology which allow 3D digital scans, taken at different epochs, be aligned and compared to quantify vertical or volume surface changes [2], [3], [4], [5]. However, there remain errors that impact on reliable quantification of change at the micron scale.

Surface registration refers to merging data files from scanned images which requires reference zones or points independently identified or operator led to create accurate alignment. Best-fit iterative-closest-point (ICP) is commonly utilised to minimise the distance between individual points within two datasets and thus quantify surface change. Its biggest advantage is its automaticity, as the registration process is led by the software’s algorithms. However, it is susceptible to errors resulting from erroneous matching of points on sequential digital files of the same surface after change or wear. This error is reduced by employing a reference-based surface registration to register scans using reference (or datum) areas, which is operator led, but finding suitable areas on teeth remains a conundrum [4].

Surface subtraction of sequential datasets has also been described in the literature [6], [7]. This refers to the software computation of the difference in a single dimension, between corresponding points from two sequential 3D surfaces producing a residual dataset that represents change and from which measurements can be taken. The residual dataset would contain a ‘step’ with data from positive or negative Z-axis heights compared to a reference zone and regardless of the original curvature of the surface [8]. Surface-subtraction metrology is particularly useful in industries assessing topographic change such as wear, corrosion, or the degree of similarity between two manufactured parts, in a relatively small area in relation to the entire sample [7]. However, one of its biggest limitations is that subtracted datasets require precise 3D alignment in the XY axes, prior to subtraction, which can introduce error and operator bias. Therefore, an automated method, combining surface-registration and surface-subtraction may offer improvements.

3D digital scans of teeth have been made by digitising stone casts with high-resolution profilometers [4], [6], [9] or laboratory scanners [10], [11], but more recently intraoral scanners has been proposed as a means of directly capturing the geometry of teeth by over sampling and stitching images together[12], [13], thus removing the need for any intermediate steps such as impression taking and cast production. Our group reported a depth measurement threshold of 44 µm for a clinical intraoral scanner in measuring wear on polished enamel [12]. However, to date, no study has described a comparable process for unpolished natural surfaces, where finding reference areas either side of a wear scar may not be possible.

Therefore, the aim of this study was to validate a novel workflow combining surface registration and subtraction to investigate the depth discrimination threshold of an intraoral scanner (IOS) on natural unpolished enamel. The null hypothesis stated that the lesion depth measurements from an intra oral scanner and a laboratory based non-contacting profilometer of unpolished freeform enamel surfaces would be the same.

## 2 Materials and methods

**Fig. 1:**
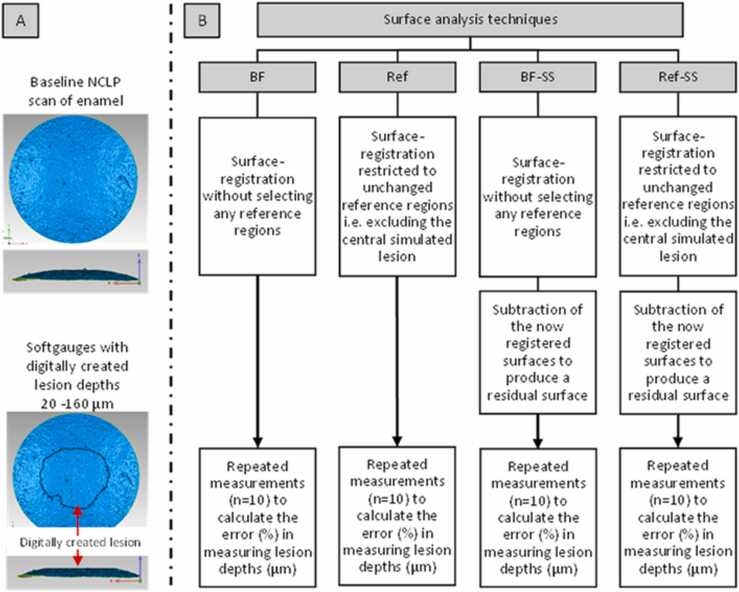
a flow diagram to show how the softgauges were used to test the accuracy of the software. The outline of the four surface-matching analysis techniques (BF, Ref, BF-SS, Ref-SS) investigated to calculate the percentage error e%) in measuring the lesions depths (20–160 µm) that were digitally created on the softgauges. BF - Best-fit surface registration, Ref - Reference-based surface-registration, BF-SS - Best-fit surface-registration and surface-subtraction, Ref-SS - Reference-based surface-registration and surface-subtraction.

This study firstly assessed, using softgauges, which combinations of software workflow performed to the highest accuracy to measure surface change on freeform surfaces (Fig. 1). Having established the most reliable combinations of surface registration and subtraction we then tested the findings on human enamel samples with increasing depths created with citric acid to determine the threshold/limits of the depth discrimination of an intra oral scanner digital scanner.

A softgauge is a digital measurement standard for testing the numerical correctness of surface metrology software [14]. A point cloud comprising XYZ cartesian coordinates from a previously existing baseline NCLP scan of a single sound natural enamel sample was manipulated in a spreadsheet software (Excel® Microsoft®, version 2008) to digitally create softgauges with known depths of 20, 40, 60, 80, and 160 µm and a consistent diameter of 1.5 mm. Four surface-matching data processing techniques were evaluated for their ability to measure the known lesion depths. Individually, a best-fit surface-registration (BF) and then a reference-based surface-registration (Ref) were tested following which best-fit surface-registration combined with surface-subtraction (BF-SS) and finally, reference-based surface-registration with subtraction (Ref-SS). Each technique was repeated ten times at the varying depths of 20, 40, 60, 80, 160 µm, based on a sample size calculation using GPower freeware, version 3.1.9 (Heinrich Heine, Dusseldorf, Germany), on previous pilot data (α error = 0.05, power = 0.80, effect size 1.30).

For the BF technique, the surface pairs (i.e., the baseline surface and the softgauge with the known lesion) consisting of Cartesian point clouds were loaded into software (Geomagic Control, 3D Systems, Darmstadt, Germany) and transformed into 3D polygon meshes. The ICP algorithm was conducted using 1500 data points to align the two meshes. For the Ref technique, the registration was restricted to unchanged reference regions, excluding the areas where surface change has occurred. For the BF-SS and Ref-SS techniques, the now registered surfaces were loaded into another software package (Mountains® 8, Digitalsurf, Besançon, France) and subtracted to produce a residual surface representing the difference between the two surfaces. Then a levelling process, utilising a best-fit-linear-least-squares plane, excluding the central area was applied. Lesion depths (μm) were reported as the mean mesh-distance (μm) between the two datasets for the BF and Ref techniques and as step-heights (μm) ISO:5436–1 (difference in height between the mid-third of the lesion and the surrounding reference area) for the BF-SS and Ref-SS techniques.

Following determination of the optimal software technique, the Ref-SS technique was used to calculate the discrimination threshold of an intra oral scanner (IOS;True Definition™, Midmark Corp., USA) on freeform surfaces and compared the data to a gold standard laboratory based non-contacting laser profilometer (NCLP;TaiCaan Technologies™, XYRIS 2000CL, UK).

Extracted sound permanent human molars were collected following ethical approval (REC ref:18/WM/0351), disinfected, then the teeth were sectioned to produce buccal/lingual samples, measuring 5 × 5 mm, and each fixed on acrylic blocks (n = 14, one sample per block). The samples were then ultrasonicated to remove contaminants and left to air-dry for 24 h prior to scanning by a single experienced operator.

The NCLP scans were conducted with a confocal displacement sensor (LT-9010 M, Keyence Corporation, Japan), employing a 655 nm-wavelength laser with a spot-diameter Ø2 μm, 600 µm vertical gauge range and 10 nm vertical resolution. Enamel surfaces were scanned using rectilinear grid-spacing at 10 µm x, y intervals, according to previously published protocols [15]. The IOS scans were conducted, following manufacturer’s instructions, after lightly coating the samples with titanium dioxide scanning spray (True Definition™ High-resolution scanning spray, Midmark Corp., USA). The IOS used freeform 3D video-scanning based on the principle of Active-Wavefront-Sampling with 6 sensors capturing 60 fps [16]. To optimise scan quality, the surface data were exported with maximal resolution (i.e., ~60 µm point-spacing).

An electrical polyvinylchloride tape with a 1.5 mm diameter circular hole, made using a punch-biopsy (BP-15 F 1.5 mm KAI Medical, Seki, Japan) was placed over the zenith of each enamel surface to provide a single protected reference region surrounding each central exposed enamel sample. A single lesion of 1.5 mm in diameter was created on each enamel sample, using a citric acid solution (1%, pH 2.2, titratable acidity 31.3 mL) at increasing immersion times, under 62.5 rpm orbital agitation (Stuart mini-Orbital Shaker SST1, Bibby Scientific, England). This resulted in 14 samples each with a single lesion with depths, at 11, 18, 23, 24, 34, 40, 56, 58, 62, 70, 73, 75, 79, and 81 µm, as measured by the gold standard NCLP. The samples were washed and air-dried for 24 h before tape removal, and then scanned again. All samples were scanned five times at baseline (T0) and post-exposure (T1) by NCLP and the IOS to create pairs (T0 + T1). Based on a priori sample size calculation requiring five T0 + T1 scans per depth level (80% power, α = 0.05, effect size 2.28 µm for NCLP vs. IOS).

Randomised pairs of scans (T0 + T1) for each sample, from the NCLP and IOS were analysed to produce residual datasets. An automated 3D surface step-height (μm) algorithm was run on all residual datasets to measure lesion depths (μm), which also auto-located and measured the XY lesion surface area (mm^2^) for comparison between the NCLP and IOS, following a previously published protocol [12]. The depth discrimination threshold of IOS was determined as the smallest depth (μm) showing no statistically significant difference to profilometry, as well as achieving 100% lesion detection (5 out of 5 analyses) based on the comparison of XY lesion surface areas (mm^2^) between IOS and NCLP. A 10% difference in area measurements was selected as an acceptable margin of error based on a previously published protocol [12].

Statistical analyses were conducted using Prism 9 (GraphPad Software Inc, California, USA). Data were checked for normality using Shapiro-Wilk test. A two-way repeated-measures ANOVA with post-hoc Bonferroni tests (p < 0.05 statistically significant) was undertaken to compare measurements between the software surface-matching analysis techniques and to compare data between IOS and NCLP measurements. Bland-Altman analysis was also used to calculate the bias and 95% limits of agreement between IOS and NCLP.

## 3 Results

Table 1 shows the differences (%) in step height from 20 to 160 µm using the softgauges with the four data workflow techniques. These data showed that the largest differences of − 29.7 to − 32.5%, were observed using BF which reduced to − 2.4 to − 3.6% in Ref technique (p < 0.0001). The combination of surface-registration and subtraction using BF-SS further reduced these differences to − 0.1 to − 0.3% (p < 0.0001) and finally 0.0% with Ref-SS (p < 0.0001) compared to BF.

**Table 1:** Mean (SD) percentage depth measuring differences (%), using softgauges at 20, 40, 60, 80 and 160 µm, for BF – Best-fit surface-registration. Ref – Reference-based surface-registration. BF-SS – Best-fit surface-registration and surface-subtraction. Ref-SS – Reference-based surface-registration and surface-subtraction. Statistical significant differences were observed between BF and Ref for all depths and BF and BF-SS and Ref-SS (p = 0.001).

	Mean (SD) depth measurement percentage error (%)			
Softgauage depth of lesion (μm)	BF	Ref	BF-SS	Ref-SS
20	-30.6 (4.8)	-3.4 (0.7)	-0.3 (0.0)	0.0 (0.0)
40	-30.1 (3.6)	-3.3 (1.5)	-0.1 (0.0)	0.0 (0.0)
60	-30.4 (1.7)	-2.4 (0.2)	-0.2 (0.0)	0.0 (0.0)
80	-29.7 (0.9)	-2.7 (0.6)	-0.1(0.0)	0.0 (0.0)
160	-32.5 (0.9)	-3.6 (1.5)	-0.1(0.0)	0.0 (0.0)

Table 2 shows step height measured by the NCLP and mean (SD) percentage step height and area (mm^2^) measurement differences for the IOS compared to NCLP using Ref-SS along with the automated lesion detection (%) of the IOS. The lesion step height ranged from 11 to 81 µm measured by the NCLP. The IOS recorded the same depths and revealed mean (SD) percentage differences in depth measurements from − 57 (14) % at the shallower depths improving to − 2 (2) % in deeper lesions. Statistically significant differences were observed between the data at depths from 18 µm (−57 (14) %, p = 0.0002) to 40 µm (−35 (9) %, p < 0.0001), and 56 µm (−17 (7) %, p = 0.0005). The mean (SD) percentage difference of IOS ranged from − 57 (14) % in shallower lesions to − 2(2) % in deeper lesions. The percentage difference of IOS for both depth and XY area measurements dropped below 10% at depths ≥ 62 µm. The automated lesion detection reveaed confidence in the depth measurement from 73 µm. No statistically significant differences were observed between the NCLP and IOS’ depth measurements above 73 µm (p > 0.05).

**Table 2:** Mean (SD) percentage measurement difference (%) of IOS for step height (µm) and area (mm^2^) compared to the NCLP, with the automated lesion detection (%) of IOS. Statistically significant differences were observed between NCLP and IOS depth measurements are shown (* = p < 0.05, ** p < 0.01, *** = p ≤ 0.001 and **** p ≤ 0.0001). Lesion depth measurements (μm) are correct to the nearest micron.

Sample number	Lesion depth (μm) NCLP	Mean (SD) depth measurement (%) of IOS vs. NCLP	Mean (SD) area measurement difference (%) of IOS vs. NCLP	Automated lesion detection (%) using IOS
1	11	-43 (41)	9 (51)	0
2	18	-57 (14) * **	-8 (28)	0
3	23	-41 (33) * **	71 (35)	0
4	24	-44 (22) * ** *	25 (11)	0
5	34	-12 (11)	-3 (26)	0
6	40	-35 (9) * ** *	3 (15)	0
7	56	-17 (7) * *	8 (4)	60
8	58	6 (6)	10 (6)	60
9	62	-6 (5)	-9 (8)	60
10	69	4 (5)	5 (5)	80
11	73	-5 (4)	2 (5)	100
12	75	-6 (3)	-5 (3)	100
13	78	-2 (2)	1 (5)	100
14	81	-4 (1)	-4 (2)	100

**Fig. 2:**
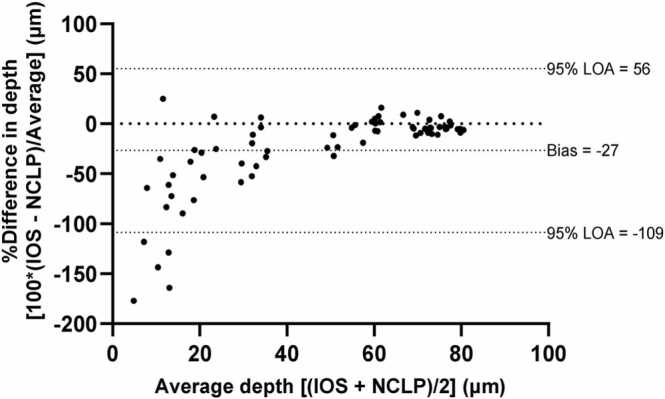
Bland-Altman plot of differences between NCLP and IOS depth measurements, expressed as percentage [100 x (IOS – NCLP)/Average)]. The overall bias was − 27% with very wide 95% Limits of Agreement (LOA) 56 to − 109%. At approximately ≥ 70 µm the % difference is near 0.

Fig. 2 shows a Bland-Altman plot of differences between the NCLP and IOS depth measurements, expressed as percentage of differences (100 x (IOS – NCLP) / average). Due to the high percentage difference between IOS and NCLP at shallower depths, the overall bias was − 27% with wide 95% Limits of Agreement (LOA), 56 to − 109%. However, as the magnitude of lesion depth increased the agreement between the two scanners increased, nearing zero percent above 70 µm lesion depths.= .

## 4 Discussion

This study demonstrated that the combination of surface-registration and subtraction significantly reduced differences for measurement of change on freeform surfaces using soft gauges. Significant differences between NCLP and IOS measurements were observed and therefore the null hypothesis was rejected. The discrimination threshold for the intraoral scanner was 73 µm above which there was confidence that any measurement was an accurate reflection of depth. These findings, although specific to this intraoral scanner, have broader impact for assessing the confidence and accuracy of any scanner used to record the surface of complex oral structures. As most intraoral scanners utilise similar physics to record the surface of teeth it is reasonable to propose that the limits around 73 µm are probable. Further work would be needed to establish criteria for each scanner, but a broad interpretation would be there are limits on their ability to distinguish varying lesion depths on teeth.

The value of the softguages was to establish which combination of software/workflow produced the optimum and most accurate results. No significant differences were observed between the two combination techniques (BF-SS and Ref-SS) suggesting that relying on reference regions alone was not necessary when a combination technique is used. This may be because the subtraction of two sequential surfaces that are well-aligned in the XY plane generates a residual ‘difference’ 3D profile of the same shape no matter the matching error in Z, hence any step height measurement thereafter on this residual surface to quantify change would be very similar. It is relatively straightforward to visualise registration errors in scans in the XY dimension. The additional step of surface subtraction eliminated errors in the Z direction, which are harder to visualise, particularly across a large 3D scan [8]. Finding reference surfaces for alignment which have not undergone changes in the oral cavity remains a significant barrier for erosion [4] and therefore the additional improvement with the surface-subtraction overcomes them. In this study, the surface-registration and subtraction combination was tested using softgauges with digitally created defects of known sizes [17]. However, it only represents differences from the software analysis and does not exclude those from hardware or operator, which can be significant; and therefore, caution needs to be applied.

Although best-fit surface-registration is relatively more straightforward and automated it has been previously demonstrated by O’Toole et al. (2018) that restricting the alignment of two 3D surfaces to unchanged reference regions minimises measurement errors and improves the accuracy of surface change [4]. However, these previous investigations were limited to techniques involving surface-registration alone, without the additional step of surface subtraction. These authors reported lower volume and vertical depth change errors in reference-based registration compared to best-fit registration and manual alignment of sequential surfaces [4]. This is because standardised best-fit algorithms are forced to draw 3D datasets into the closest mathematical proximity possible, in a way that is not biologically informed of the lesion location, often resulting in inaccurate lesion quantification that limits diagnostic potential. Restricting these algorithms to surfaces that are least likely to have undergone change means they are less susceptible to outliers and results in a more accurate analysis [4], [18]. Mylonas et al. (2019) investigated the use of surface-subtraction in characterising early erosion on natural enamel, however, they used a physical positioning jig to ensure repeatable placement of each enamel sample [6], which is a less automated.

Once the combination of surface registration and subtraction was validated, it was applied to sequential scans to show the threshold of the IOS on measuring lesions on unpolished enamel. In contrast to the intraoral scanner, the high-resolution NCLP detected lesions of all simulated depths. Shallow lesion depths were particularly challenging for the IOS to measure accurately. This was highlighted by the wide standard deviations in depth and area measurements, poor automated lesion area detection in shallow lesions, as well as the Bland-Altman plot which showed wide limits of agreement and a clear trend of improved percentage differences between the two devices as the lesion depth increased.

The data showed that as the depth increased the percentage difference compared to the NCLP and the standard deviations reduced. The resolution of the intraoral scanner at the lower values of the lesion depth was insufficient to discriminate an accurate value and both the step height and the area measurement had variation. This was confirmed by the automated lesion detection which is a previously published method to identify the point at which the image seen on the scans taken by the IOS became clear [12]. As the depth increased the difference reduced and the lesion detection followed the same trend. At 73 µm the step height and area had reduced to minimal levels and the lesion detection was 100%. This showed there was high confidence that the intra oral scanner was able to discriminate and so measure the lesion depth.

Unlike point-measuring high-resolution scanners, such as profilometers, which can sharply focus an optical beam onto a surface, intraoral scanners capture surface features and their optical interactions over an area simultaneously. They achieve this by oversampling and averaging multiple points of measurement representing the same area [19]. Although intraoral scanners capture surface topography significantly faster than profilometers, their drawback is lower spatial resolution (lower point cloud density) and point measuring accuracy. The impact of lower spatial resolution results in surfaces with smoother topography and poorer depiction of the margins of the lesion [12]. The addition of surface-subtraction following registration enabled the IOS to reliably quantify surface change on unpolished enamel at 73 µm, a depth that has either not previously been tested nor demonstrated agreement as high as this present study. Though, the underlying constraints associated with the physics of sampling remained and limit the threshold.

A number of in vitro and in vivo studies utilising intraoral scanners for wear quantification have published, most of which do not compare data to a gold standard [20], [21], [22], [23], [24], [25]. Any IOS has limitations because of the way the profiles are formed. Recently, quantitative agreement was demonstrated between an intraoral scanner and micro-CT volumetric and depth wear measurements [26]. Hartkamp et al. (2017) were the first to use a profilometer compared to an intraoral scanner to measure vertical wear which suggested an agreement within approximately 20 µm; however, the depths were over 70 µm while lateral measurements of the simulated lesions were not specified [23]. A different intraoral scanner was utilised in this present study to assess surface change as small as 11 µm depth. Furthermore, IOS surface change analysis was not restricted to measuring vertical tissue loss (μm); instead, the XY lesion area measurements (mm^2^) were compared against profilometry in order to further scrutinize IOS’s performance.

The authors have previously reported the threshold for measuring lesions on polished enamel to be 44 µm using the same IOS [12]. This is lower than the measurement threshold of IOS in this study, 73 µm. This probably reflects that polished enamel has a simpler and flatter morphology, but more importantly, it allows single-scan analysis, utilising reference areas around the lesion to measure tissue loss. Furthermore, simulated lesions on polished enamel have more distinct boundaries which would favour an automated system. The relative performance of the multi-step process required for measuring change on complex freeform surfaces is more prone to measurement uncertainty as each sequential scan introduces an individual set of errors [10] while the alignment process, which is itself dependant on the scanner’s accuracy and resolution, is a major contributor of measurement error [4].

Addressing the limitations of the study, a single experienced operator and two software packages tested the different data processing techniques. The impact of other operators and different software needs further investigation, but manipulation of the workflow needs training and experience. Although automation was used whenever possible some degree of operator judgement is still necessary. The software which is used to superimpose and subtract are complex mathematical algorithms and are hidden from the operator. The authors have to rely on the workflows and whilst we did our best to understand and test the conditions these are commercial products. The biggest drawback of the proposed surface-registration and subtraction technique is its time-consuming and cumbersome process, having to use two different software, requiring roughly double the analysis time than a stand-alone surface-registration technique without the subtraction step. Therefore, further work is needed on automating the workflow and making it more suitable for diagnosis in clinical care.

In conclusion, surface-subtraction after -registration improves the accuracy of measuring surface change on sequential scans. This has potential to improve diagnostics in many fields of dentistry. Using this technique, the intraoral scanner was able to predictably determine changes of 73 µm, a level of accuracy that may be acceptable for future studies and clinical monitoring of surface changes over time such as tooth or material wear.

## Funding

This project was funded via a Medical Research Council Industrial Collaborative Award in Science and Engineering (UK MRC-iCASE) studentship (MR/R015643/1), for which Glaxo-SmithKline Consumer Healthcare Oral Health Research & Development was the industrial collaborator. The funding sources were not involved in the collection, analysis, and interpretation of data; in the writing of the report; or in the decision to submit the article for publication. They read the manuscript prior to submission.

## CRediT authorship contribution statement

**Polyvios Charalambous**: Contributed to conception, design, data acquisition, analysis and interpretation, Performed all statistical analyses, Drafted and critically revised the manuscript. **Saoirse O’Toole**: Contributed to conception, Data interpretation and critically revised the manuscript. **Rupert Austin**: Contributed to conception, design, data analysis and interpretation, Critically revised the manuscript. **David Bartlett**: Contributed to conception, design, data interpretation, Critically revised the manuscript. All authors gave their final approval and agree to be accountable for all aspects of the work.
